# Comparative Impacts of Oral Amoxicillin, Azithromycin, and Clindamycin on Gut Microbiota and Intestinal Homeostasis

**DOI:** 10.3390/antibiotics15010024

**Published:** 2025-12-25

**Authors:** Shanshan Li, Jing Sun, Yanfang Ren, Songlin Wang

**Affiliations:** 1 School of Stomatology, Henan University, Zhengzhou 450046, China; shanshanli@henu.edu.cn; 2 Laboratory of Oral Health and Homeostatic Medicine, School of Stomatology and Beijing Laboratory of Oral Health, Capital Medical University, Beijing 100070, China; sunjing6721@163.com; 3 Central Laboratory, Jinan Key Medical and Health Laboratory of Oral Diseases and Tissue Regeneration, Jinan Key Laboratory of Oral Diseases and Tissue Regeneration, Shandong Provincial Key Medical and Health Laboratory of Oral Diseases and Tissue Regeneration, Jinan Stomatological Hospital, Jinan 250001, China; 4 Department of Biochemistry and Molecular Biology, School of Basic Medical Sciences, Capital Medical University, Beijing 100069, China; 5 Eastman Institute for Oral Health, University of Rochester Medical Center, 625 Elmwood Ave., Rochester, NY 14620, USA; 6 Laboratory of Oral Homeostatic Medicine, School of Medicine and SUSTech Homeostatic Medicine Institute (SHMI), Southern University of Science and Technology, Shenzhen 518055, China

**Keywords:** amoxicillin, azithromycin, clindamycin, gut microbiota

## Abstract

**Background**: Amoxicillin, clindamycin and azithromycin are the most frequently prescribed antibiotics for odontogenic infections, but their comparative effects on gut microbiota and intestinal homeostasis remain insufficiently understood. Disruption of gut microbiota, short-chain fatty acid (SCFA) production, and mucosal barrier integrity may contribute to gastrointestinal symptoms. We aimed to compare the impacts of these antibiotics on gut microbiota, SCFA levels, and colonic goblet cells. **Methods**: C57BL/6N mice were treated with oral amoxicillin, clindamycin, or azithromycin at clinically relevant dosages. Cecal index, fecal water content, and diarrhea index were assessed during treatment and recovery. Gut microbiota composition and absolute bacterial abundance were determined using 16S rRNA amplicon absolute quantification sequencing. SCFAs in cecal contents were quantified by gas chromatography–mass spectrometry. Goblet cell abundance and *Muc2* mRNA expression in colon tissues were evaluated using Alcian blue staining and RT-PCR. **Results**: Amoxicillin caused moderate increases in cecal index, reduced *Ligilactobacillus* abundance, increased *Escherichia-Shigella*, lowered SCFA levels, and decreased goblet cells and *Muc2* expression, with partial recovery after two weeks. Clindamycin induced more severe dysbiosis, including sustained Proteobacteria expansion, persistent loss of beneficial taxa, 86–90% reduction in SCFA production, and lasting decreases in goblet cells and *Muc2* expression without recovery during the observation period. Azithromycin caused mild and reversible changes across all parameters. **Conclusions**: Among the three antibiotics, azithromycin had the least detrimental effects on gut microbiota, SCFA production, and mucosal barrier function, whereas clindamycin caused profound and persistent intestinal disruption. These findings provide comparative evidence to inform antibiotic selection in clinical practices.

## 1. Introduction

Dental caries and periodontitis are the most prevalent bacterial infectious diseases worldwide, posing significant public health burdens across age groups and populations [1,2]. Systemic antibiotics are frequently prescribed in dentistry to manage conditions, such as acute periapical abscesses and acute periodontal infections, and to prevent complications in medically vulnerable individuals, including patients with infective endocarditis risk, prosthetic heart valves, or recent joint replacements. Dental professionals account for approximately 10–20% of all antibiotic prescriptions in healthcare settings [3,4,5]. However, even short treatment courses and very low antibiotic dosages can disrupt the intestinal microbiota [6,7], reduce microbial diversity, and promote dysbiosis with potential consequences for gastrointestinal and systemic health [8,9]. Such alterations may also increase the abundance of antibiotic resistance genes, contributing to adverse clinical outcomes, prolonged hospitalization, and reduced host resilience against pathogens [10].

Multiple antibiotic classes are used for odontogenic infections, including penicillins, clindamycin, macrolides, cephalosporins, metronidazole, and tetracyclines. Amoxicillin is a broad-spectrum β-lactam with potent activity against Gram-negative organisms, and the most commonly prescribed antibiotic in dental practice worldwide due to its efficacy and pharmacokinetic profile. For patients who are allergic to penicillin, clindamycin has historically been widely used as an alternative in many countries [11,12,13,14,15,16,17,18,19,20] due to its excellent oral absorption, penetration into bone and soft tissues, and high intra-tissue concentrations within the infection site [21]. Azithromycin, a macrolide with an antibacterial spectrum similar to penicillin and a long half-life, is also used as an alternative for individuals allergic to penicillin [17,22,23,24], supported by clinical guidance recommending its use in selected cases [21,25].

Antibiotic exposure can disrupt intestinal homeostasis by reducing microbial diversity, changing taxonomic composition, and decreasing production of short-chain fatty acids (SCFAs) [26,27]. SCFAs are critical for intestinal epithelial cell energy metabolism, anti-inflammatory signaling, and maintenance of mucosal barrier integrity [28]. Antibiotics may also impair goblet cell function and mucin production, particularly *Muc2*, which forms the dominant protective mucus barrier separating gut bacteria from the colonic epithelium [29,30,31]. Disruption of these host–microbe interactions compromises epithelial integrity, increases inflammatory susceptibility, and may exacerbate post-antibiotic gastrointestinal symptoms.

Given the frequent use of amoxicillin, clindamycin, and azithromycin in dental practice, a comparative evaluation of their impacts on gut microbiota and homeostasis is clinically relevant. We aimed to study the differential impacts of these three antibiotics at clinically relevant doses on gut microbial composition, SCFA concentrations, and colonic goblet cells in C57BL/6N mice. By characterizing antibiotic-specific patterns of dysbiosis and mucosal disturbance, this study can provide experimental evidence to inform antibiotic stewardship and guide safer therapeutic selection in clinical practices.

## 2. Results

### 2.1. Effects of Antibiotics on Cecal Index, Fecal Water Content, and Diarrhea Index

**Figure 1:**
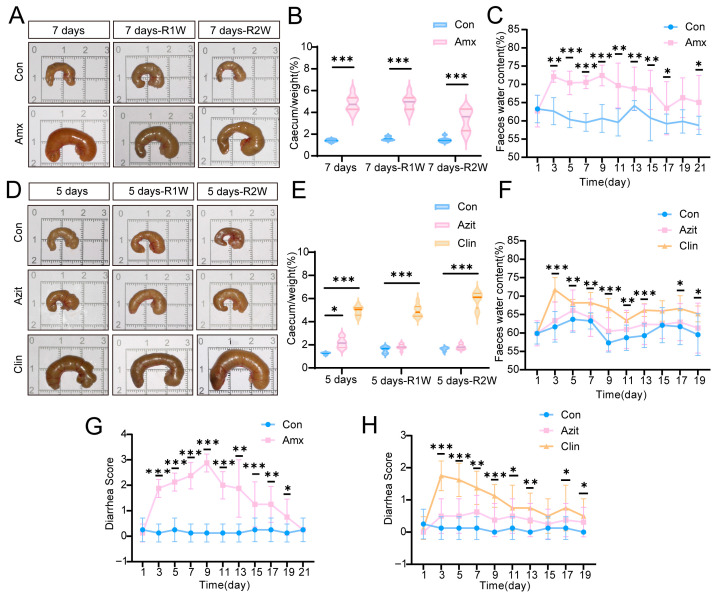
Effects of Antibiotics on Cecal Index, Fecal Water Content, and Diarrhea Index in C57BL/6N Mice. Representative cecal samples from C57BL/6N mice treated with amoxicillin (**A**), clindamycin (**D**), or azithromycin (**D**) were examined at various time points. Cecal index in mice treated with amoxicillin (**B**), azithromycin, and clindamycin (**E**) over time. The cecal index was significantly elevated. In the recovery phase, the cecal index of the amoxicillin group showed a slight decrease at two weeks compared to one-week post-treatment, whereas that of the clindamycin group exhibited a slight increase at two weeks relative to one week. The cecal index in the azithromycin group increased after antibiotic administration but returned to baseline levels during the recovery periods. Fecal water content in mice after administration of amoxicillin (**C**), azithromycin, and clindamycin (**F**) at different time intervals. Diarrhea index in mice treated with amoxicillin (**G**), azithromycin, and clindamycin (**H**) across time points. In the amoxicillin and clindamycin groups, fecal water content and the diarrhea index were observed to increase significantly starting from the third day. Abbreviations: Con, control group; Amx, amoxicillin group; Azit, azithromycin group; Clin, clindamycin group. 7 days: 7 days of antibiotic administration; 7 days–R1W: 7 days of antibiotic administration followed by 1 week of recovery; 7 days–R2W: 7 days of antibiotic administration followed by 2 weeks of recovery; 5 days: 5 days of antibiotic administration; 5 days–R1W: 5 days of antibiotic administration followed by 1 week of recovery; 5 days–R2W: 5 days of antibiotic administration followed by 2 weeks of recovery. Data are presented as mean ± SD (*n* = 8). Statistical significance: (**B**,**C**,**E**,**G**) * *p* < 0.05, ** *p* < 0.01, *** *p* < 0.001 compared with the control group. (**F**,**H**) * *p* < 0.05, ** *p* < 0.01, *** *p* < 0.001, clindamycin group vs. control group.

Significant differences in the cecal index were observed in mice treated with amoxicillin (Figure 1A,B) and clindamycin (Figure 1D,E) compared to the control group at three time points (*p* < 0.001). The cecal index of mice treated with amoxicillin decreased at the two-week recovery time point compared to the one-week recovery time point, whereas the cecal index of mice treated with clindamycin increased during the same period. In contrast, the cecal index of mice treated with azithromycin showed a significant difference only at the five-day time point, returning to baseline levels during the recovery period (Figure 1D,E).

Fecal water content in mice treated with amoxicillin was significantly higher on day 3 compared to the control group, remaining elevated throughout the experiment, decreasing from 73% to 65% (Figure 1C). Consistent with this trend, the diarrhea index in the amoxicillin group was significantly higher than that in the control group from day 3 to day 19 (Figure 1G). The clindamycin-treated group exhibited a similar trend in fecal water content to the amoxicillin group (Figure 1F), while no significant differences were observed in the azithromycin group compared to the control group (Figure 1F). The diarrhea index in the clindamycin group followed a pattern similar to the amoxicillin group, while the azithromycin group showed slightly improved outcomes relative to the clindamycin group (Figure 1H).

### 2.2. Effect of Antibiotics on the Fecal Microbiota of Mice

#### 2.2.1. Effect of Various Antibiotics on the Alpha Diversity of Mice

Alpha diversity, which signifies the biodiversity within a specific region or ecosystem, reflects the variety of species within a given sample. It is commonly assessed using diversity indices based on species richness or evenness. The Chao1 index is frequently utilized to estimate the total number of species in a community, where higher values indicate greater species richness. The Shannon index offers an objective measure of species diversity, with higher values indicating increased diversity. Pielou’s Evenness index gauges the ratio of the observed Shannon diversity index to its maximum potential value, assessing the evenness of species distribution within a community. A value close to 1 indicates a nearly uniform species distribution, while a value nearing 0 suggests domination by a few species within the community.

**Figure 2:**
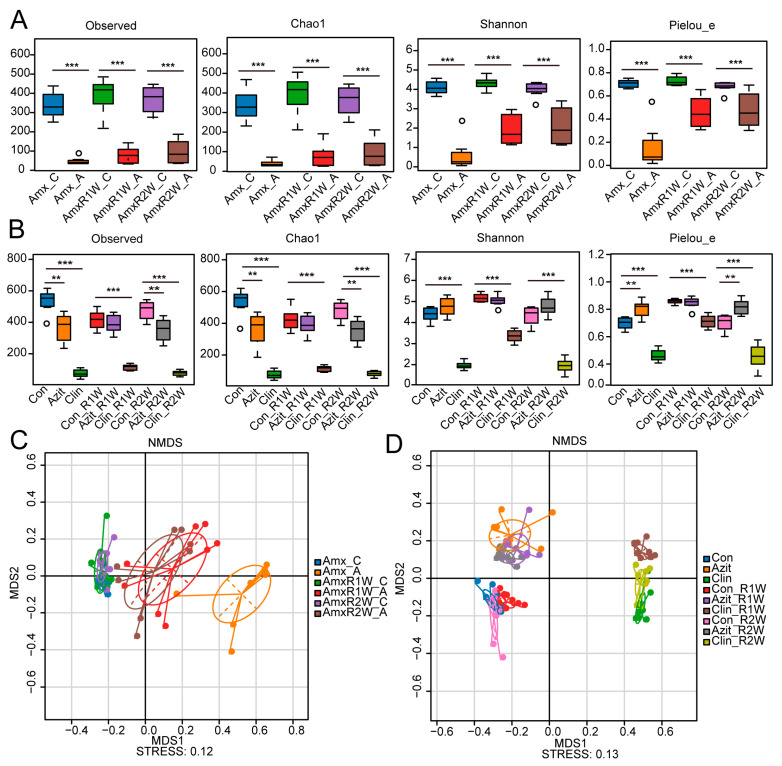
The Effects of Amoxicillin, Azithromycin, and Clindamycin on the Diversity of Gut Microbiota in C57BL/6N Mice. Alpha diversity analysis was performed using the observed species index, Chao1, Shannon and Pielou’s Evenness index for the amoxicillin group (**A**), azithromycin group, and clindamycin group (**B**) by Accurate 16S Absolute Quantification Sequencing. Beta-diversity analysis was conducted through non-metric multi-dimensional scaling (NMDS) ordination based on Bray–Curtis dissimilarity of fecal sample community compositions from the amoxicillin group (**C**), azithromycin group, and clindamycin group (**D**). The fecal samples from the Amoxicillin and Azithromycin groups, collected one week and five days post-treatment, respectively, exhibited distinct differences from the control group, with the compositional dissimilarity gradually decreasing over time. In contrast, the beta-diversity distance in the clindamycin group remained relatively stable throughout the observation period. Abbreviations: Amx_C: Group with 7 days of 0.9% NaCl solution administration; Amx_A: Group with 7 days of amoxicillin administration; AmR1W_ C: Group with 7 days of 0.9% NaCl solution administration followed by 1 week of recovery; AmR1W_ A: Group with 7 days of amoxicillin administration followed by 1 week of recovery; AmR2W_ C: Group with 7 days of 0.9% NaCl solution administration followed by 2 weeks of recovery; AmR2W_ A: Group with 7 days of amoxicillin administration followed by 2 weeks of recovery; Con: Group with 5 days of 0.9% NaCl solution administration; Azit: Group with 5 days of azithromycin administration; Clin: Group with 5 days of clindamycin administration; Con_R1w: Group with 5 days of 0.9% NaCl solution administration followed by 1 week of recovery; Azit_R1w: Group with 5 days of azithromycin administration followed by 1 week of recovery; Clin_R1w: Group with 5 days of clindamycin administration followed by 1 week of recovery; Con_R2w: Group with 5 days of 0.9% NaCl solution administration followed by 2 weeks of recovery; Azit_R2w: Group with 5 days of azithromycin administration followed by 2 weeks of recovery; Clin_R2w: Group with 5 days of clindamycin administration followed by 2 weeks of recovery. The data is presented as mean ± SD (*n* = 8), with statistical significance denoted as ** *p* < 0.01, *** *p* < 0.001 compared with the control group. The circles outside the bars represent outlier values.

In the amoxicillin group, significant differences were noted between the antibiotic-treated group and the control group at three distinct time points (*p* < 0.001) for the observed species index, Chao1, Shannon, and Pielou’s evenness index. The average values displayed a slight upward trend over time (Figure 2A). Similarly, in the clindamycin group, significant variances were observed between the antibiotic-treated and control groups at all three time points for the same diversity indices. However, these indices remained relatively stable throughout the recovery period. In the azithromycin group, significant differences were observed for the observed species index, Chao1, and Pielou’s evenness index at 5 days and two weeks post-administration. In contrast, no significant difference was detected in the Shannon index between the azithromycin-treated and control groups across the three time points (Figure 2B).

#### 2.2.2. Effect of Different Antibiotics on the Beta-Diversity of Mice

Beta-diversity was assessed using non-metric multidimensional scaling (NMDS) ordination, utilizing Bray–Curtis dissimilarity of fecal microbial community. In the amoxicillin group, fecal samples collected after one week of treatment showed distinct differences from the control group. Over time, the compositional distance between the recovery groups and the control group decreased gradually, with the one-week recovery group displaying a reduced distance compared to the one-week treatment group, and the shortest distance observed in the two-week recovery group (Figure 2C). A similar trend was observed in the azithromycin-treated group, while in the clindamycin-treated group, the Beta-diversity distance remained relatively stable throughout the observation period (Figure 2D).

#### 2.2.3. Effect of Various Antibiotics on the Fecal Microbiome Composition in Mice at Phylum and Genus Level

**Figure 3:**
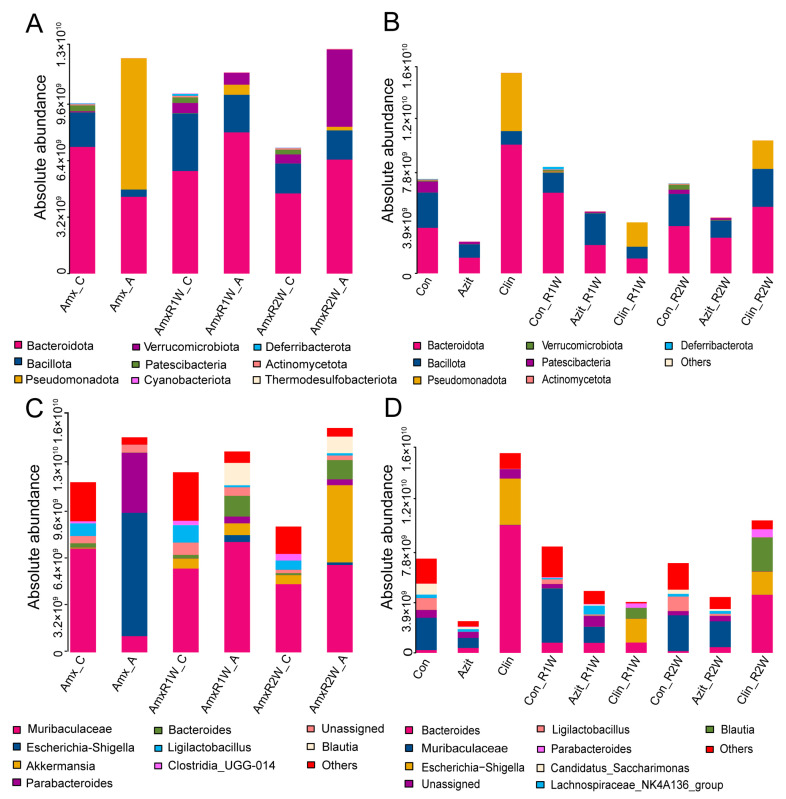
The Composition of the Fecal Microbial Community in C57BL/6N Mice Treated with Amoxicillin, Azithromycin, and Clindamycin at the Phylum and Genus Levels. The bar plot shows the absolute abundance of the microbial community composition at the phylum (**A**,**B**) and genus (**C**,**D**) level in the amoxicillin group, azithromycin group, and clindamycin group at different time points, as analyzed by Accurate 16S absolute quantification sequencing. The fecal microbial community composition in the Amoxicillin group tended to normalize during the recovery period, while no significant recovery was observed in the clindamycin group. In contrast, the composition in the azithromycin group resembled that of the control group. The bar plot illustrates the average absolute abundance of bacterial taxa at the phylum and genus levels *(n* = 8).

Following amoxicillin treatment, the absolute abundance of Pseudomonadota in the Amx_A group was 7.4 × 10^9^ copies/g feces, constituting 60.96% of the microbiome. In contrast, the Amx_C group showed a lower abundance of Pseudomonadota at 1.1 × 10^7^ copies/g feces, representing 3.43%. Subsequent groups (AmxR1W_A and AmxR2W_A) displayed further decreases in Pseudomonadota abundance to 5.05% and 1.57%, respectively. Bacillota levels increased from 3.33% in the Amx_A group to 18.66% and 13.03% in the AmxR1W_A and AmxR2W_A groups, respectively. The relative abundance of Verrucomicrobiota peaked at 34.4% in the AmxR2W_A group (Figure 3A and Figure S1A–F).

In the azithromycin-treated group, fecal microbial abundance decreased post-antibiotic treatment but gradually recovered over time compared to the control group. Conversely, both the Clin group and Clin_R2W group exhibited increased fecal microbial abundance compared to the control group. Significant changes in microbial composition were noted in the clindamycin-treated group, with Pseudomonadota abundance at 4.50 × 10^9^ copies/g feces (28.97% in the Clin group), rising to 47.79% in the Clin_R1W group, and then decreasing to 21.33% in the Clin_R2W group, remaining higher than the control group (Figure 3B and Figure S1G–O).

At the genus level, the absolute abundance of *Escherichia–Shigella* was 7.0 × 10^9^ copies/g feces, accounting for 57.34% in the Amx_A group, which was significantly higher than that in the Amx_C group (7.0 × 10^6^ copies/g feces, accounting for 0.08%). However, its absolute abundance was markedly reduced in the AmxR1W_A and AmxR2W_A groups, with values of 3.90 × 10^8^ and 1.45 × 10^8^ copies/g feces, respectively. In contrast, the absolute abundance of *Muribaculaceae* was 9.21 × 10^8^ copies/g of feces in the Amx_A group, which increased to 6.72 × 10^9^ and 4.96 × 10^9^ in the AmxR1W_A and AmxR2W_A groups, respectively—values that were comparable to those observed in the control group. Notably, *Akkermansia* was most abundant in the AmxR2W_A group (4.38 × 10^9^ copies/g feces, 34.41% of microbial composition), being the predominant genus among all amoxicillin-treated groups (Figure 3C).

The fecal microbial community in azithromycin-treated mice resembled the control group, while a distinct profile was seen in the clindamycin-treated group. *Escherichia–Shigella* abundance in the Clin group was 3.59 × 10^9^ copies/g feces (11.6% of total population), decreasing to 1.85 × 10^9^ and 1.79 × 10^9^ copies/g feces in the Clin_R1W and Clin_R2W groups, respectively. Similarly, *Bacteroides* abundance in the Clin group was 9.94 × 10^9^ copies/g feces, declining to 8.04 × 10^8^ and 4.51 × 10^9^ copies/g feces in the Clin_R1W and Clin_R2W groups, respectively (Figure 3D).

#### 2.2.4. Effects of Various Antibiotics on the Differentially Expressed Key Bacterial Genera in the Fecal Microbiota

**Figure 4:**
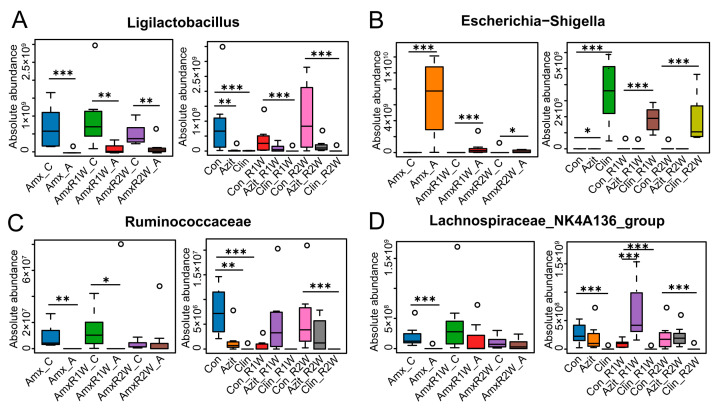
Key Bacterial Genera in the Fecal Microbiota of C57BL/6N Mice After Treatment with Amoxicillin, Azithromycin, and Clindamycin. Boxplots depict the average absolute abundance of key bacterial genera that exhibited significant variation across samples from the respective treatment groups by Accurate 16S Absolute Quantification Sequencing. Amoxicillin dramatically reduced the absolute abundance of *Ligilactobacillus* during treatment and showed a slight increase during the recovery period. Azithromycin also decreased the level of *Ligilactobacillus* in the Azit group but restored it to normal levels during the recovery period. Conversely, clindamycin led to a substantial reduction in *Ligilactobacillus* levels throughout the experimental period (**A**). Similar trends were observed for *Ruminococcaceae* (**C**) and the *Lachnospiraceae_NK4A136_group* (**D**). Amoxicillin significantly increased the absolute abundance of *Escherichia-Shigella* in the Amx_A group (**B**). Azithromycin reduced *Escherichia-Shigella* levels in the Azit group compared to the control, tending towards normalization during recovery. However, in the clindamycin-treated group, the level of *Escherichia-Shigella* significantly increased and remained at a relatively high level. The data is presented as mean ± SD (*n* = 8). * *p* < 0.05, ** *p* < 0.01, *** *p* < 0.001 compared with the control group. The circles outside the bars represent outlier values.

Several differentially expressed key bacterial genera were identified through 16S rRNA absolute quantification sequencing following antibiotic administration, including *Ligilactobacillus* (Figure 4A), *Escherichia-Shigella* (Figure 4B), *Ruminococcus* (Figure 4C), *Lachnospiraceae_NK4A136_group* (Figure 4D), and others (Figure S2). Amoxicillin significantly reduced the absolute abundance of *Ligilactobacillus* to 4.98 × 10^6^ copies/g feces. However, a slight increase was observed in the Amoxicillin recovery groups, with abundance rising from 1.10 × 10^8^ copies/g feces in the AmxR1W_A group to 1.28 × 10^8^ copies/g fecal in the AmxR2W_A group. In azithromycin-treated mice, the *Ligilactobacillus* level was 1.27 × 10^7^ copies/g feces in the Azit group, showing a significant difference compared to the control. However, during the recovery period, no significant differences were observed between the Azit_R1W and Azit_R2W groups, with levels of 9.52 × 10^7^ and 1.55 × 10^8^ copies/g feces, respectively. In contrast, in the clindamycin-treated group, the level of *Ligilactobacillus* remained low, ranging from 8.13 × 10^6^ to 2.18 × 10^6^ copies/g feces, with no apparent recovery over time. Similar trends were observed for *Ruminococcaceae* (Figure 4C) and the *Lachnospiraceae_NK4A136 group_group* (Figure 4D).

For *Escherichia-Shigella* (Figure 4B), Amoxicillin significantly increased the absolute abundance to 7.0 × 10^9^ copies/g feces in the Amx_A group compared to 7.49 × 10^6^ copies/g feces in the Amx_C group. However, the abundance decreased dramatically from 3.90 × 10^8^ copies/g fecal in the AmxR1W_A group to 1.45 × 10^8^ copies/g feces in the AmxR2W_A group. But in azithromycin-treated mice, azithromycin was found to reduce the level of *Escherichia-Shigella* to 8.33 × 10^5^ copies/g feces in the Azit group compared to the control group, with no significant differences observed during the recovery period. However, in the clindamycin-treated group, the level of *Escherichia-Shigella* dramatically increased to 3.59 × 10^9^ copies/g feces in the Clin group, and remained at a relatively high level, ranging from 1.85 × 10^9^ copies/g feces to 1.19 × 10^9^ copies/g feces in the Clin_R1W and Clin_R2W groups, respectively, indicating no apparent recovery over time.

### 2.3. Effects of Amoxicillin, Azithromycin, and Clindamycin on the SCFAs in C57BL/6N Mice

**Figure 5:**
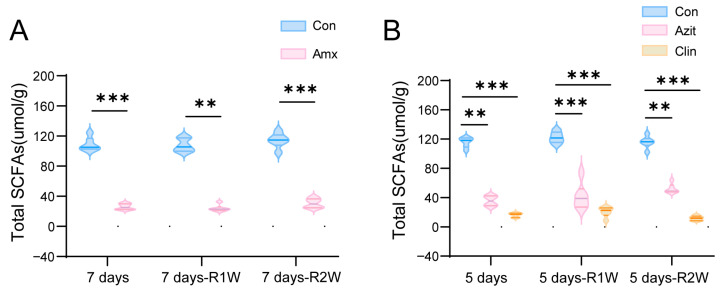
Effects of Amoxicillin, Azithromycin, and Clindamycin on the SCFAs in C57BL/6N Mice. GC-MS analysis was conducted on cecal content from these mice post-administration of the antibiotics. Amoxicillin led to a significant decrease in total SCFA levels, with a partial recovery observed over time (**A**). Azithromycin treatment resulted in notably lower total SCFA levels compared to the control groups. After a two-week recovery period, a 55% reduction in total SCFA levels was noted, less severe than the initial 70% decrease after five days of treatment. Conversely, the clindamycin group exhibited a marked and consistent decline in SCFA levels across all time points, surpassing the reductions seen in the amoxicillin and azithromycin groups (**B**). The abbreviations used are as follows: Con for control group, Amx for amoxicillin group, Azit for azithromycin group, Clin for clindamycin group, and SCFAs for short-chain fatty acids. Data is presented as mean ± standard deviation (*n* = 6). Significance levels are denoted as, ** *p* < 0.01, *** *p* < 0.001 compared to the control group.Dotted horizontal line at y = 0: baseline for distinguishing positive and negative values.

Amoxicillin was found to decrease the levels of acetic acid, propionic acid, isobutyric acid, butyric acid, isovaleric acid, valeric acid, and total SCFAs in cecal contents at various time points, with the exception of isovaleric acid in the AmxR2W group (Figure 5A and Figure S3). After one week of amoxicillin treatment, total SCFAs dropped to 24%—with a slight rise to 27% observed following a two-week recovery period. In mice treated with azithromycin, the levels of acetic acid, propionic acid, isobutyric acid, and total SCFAs were significantly lower than those in their respective control groups. After a two-week recovery period, azithromycin reduced total SCFA levels by 55% relative to the control group—a less pronounced decrease than the 70% reduction observed five days post-treatment. In contrast, clindamycin treatment consistently decreased SCFA levels by 86% to 90% across all time points, indicating a strong and sustained impact on SCFA production (Figure 5B and Figure S3).

### 2.4. Effects of Antibiotics on the Intestinal Mucosal Barrier

**Figure 6:**
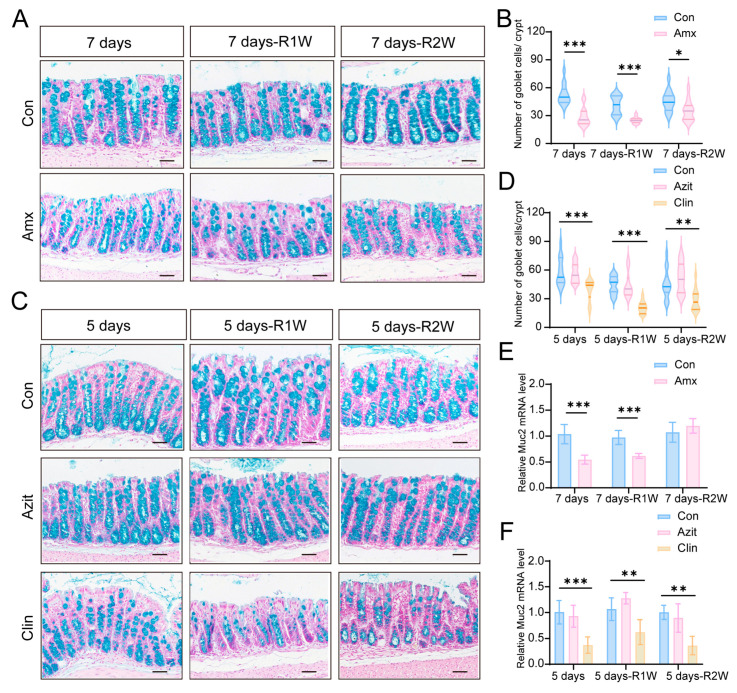
The Effects of Amoxicillin, Azithromycin, and Clindamycin on Colon Tissue. Alcian Blue staining was performed to evaluate the impact of these antibiotics on goblet cells in the distal colon tissue. Amoxicillin treatment led to a decrease in the number of goblet cells per crypt, with a slight restoration observed after a two-week recovery period (**A**,**B**). No significant changes in the number of goblet cells per crypt were observed in the azithromycin-treated group. In contrast, Clindamycin treatment significantly decreased the number of goblet cells per crypt, with no observed restoration after a two-week recovery (**C**,**D**). mRNA expression levels of *Muc2* in colon tissues were assessed using RT-PCR. Amoxicillin treatment led to a significant reduction in *Muc2* mRNA levels after one week, which returned to normal after a two-week recovery (**E**). No significant changes in *Muc2* mRNA expression were observed in mice treated with azithromycin. Clindamycin treatment markedly decreased *Muc2* mRNA levels, with no observed restoration even after a two-week recovery (**F**). Scale bars: 50 µm. Con denotes the control group; Amx, the amoxicillin group; Azit, the azithromycin group; Clin, the clindamycin group. Data is expressed as mean ± standard deviation (*n* = 5). * *p* < 0.05, ** *p* < 0.01, *** *p* < 0.001 compared with the control group.

To assess the impact of amoxicillin, azithromycin, and clindamycin on the goblet cells in distal colon tissue, Alcian Blue staining was performed. Following one week of amoxicillin treatment, the number of goblet cells per crypt in the colon of mice decreased by 47% compared to the control group. However, after a two-week recovery period, this reduction partially reversed, with the amoxicillin-treated group showing a 39% lower count of goblet cells per crypt compared to the control group (Figure 6A,B). In contrast, no significant difference was observed in goblet cell count between azithromycin-treated mice and the control group. Conversely, mice treated with clindamycin for five consecutive days exhibited a 34% decrease in goblet cells per crypt in the colon compared to the control group. Unexpectedly, this reduction did not improve during the two-week recovery period but instead worsened, with the clindamycin-treated group showing a 53% lower count of goblet cells per crypt compared to the control group (Figure 6C,D).

Furthermore, we assessed the expression levels of *Muc2* using RT-PCR to determine the mRNA expression of *Muc2* in colon tissues after the administration of various antibiotics. Consistent with the observed changes in goblet cell expression patterns, the mRNA level of the *Muc2* gene in colon tissues from amoxicillin-treated mice decreased by 46%, but returned to normal after a two-week recovery period (Figure 6E). In contrast, no significant change in *Muc2* mRNA expression was detected in azithromycin-treated mice. However, clindamycin treatment led to a substantial reduction in *Muc2* mRNA levels in colon tissues five days post-administration, with no recovery observed even after two weeks. At the end of the observation period, *Muc2* mRNA levels remained 64% lower in the clindamycin-treated group compared to the control group (Figure 6F).

## 3. Discussion

The findings of this study indicate that amoxicillin, azithromycin, and clindamycin have significantly different effects on gut microbiota and homeostasis. Azithromycin caused only mild and reversible changes, amoxicillin induced moderate but partially recoverable disturbances, and clindamycin produced profound and persistent dysbiosis. These differences were consistently reflected across cecal index enlargement, alterations in microbiota structure and absolute abundance, reductions in SCFAs, and impairment of goblet cell function and *Muc2* expression. Together, these results highlight that clindamycin has the most substantial detrimental impact on intestinal ecology, whereas azithromycin exhibits the most favorable intestinal safety profile.

The antibiotic-specific patterns observed in this study align with growing evidence that different drug classes vary substantially in their propensity to disrupt the gut microbiome. Amoxicillin and clindamycin both reduced α-diversity and altered β-diversity, whereas azithromycin produced milder compositional shifts.

Consistent with previous clinical and experimental work, amoxicillin caused expansion of Proteobacteria, particularly *Escherichia-Shigella*, and depletion of *Ligilactobacillus*, but these changes partially normalized within two weeks. These findings are in agreement with previous studies that reported transient reductions in diversity and butyrate-producing taxa following amoxicillin, with recovery periods depending on dose and duration [8]. Multiple clinical trials on amoxicillin have demonstrated that its administration can decrease the α-diversity of the intestinal microbiota [32], reduce the levels of SCFAs [33,34], and increase the abundance of *Escherichia coli/Shigella* [35], aligning with our study’s results. Recovery of intestinal flora post-amoxicillin treatment varies across studies. One clinical study reported that both the intestinal microbiota and antibiotic-resistant microbial populations returned to baseline levels within one week after the discontinuation of short-term amoxicillin treatment [36]. In contrast, another study suggested that the microbial composition generally recovered to baseline within approximately 2 to 4 weeks after amoxicillin administration [37]. Moreover, certain significant alterations induced by amoxicillin were observed to persist for up to 6 weeks post-treatment [35]. The observed discrepancies in clinical findings may be attributed to variations in the specific penicillin antibiotics used, dosage regimens, treatment duration, and microbial analysis methods.

Clindamycin, in contrast, caused the most significant and most persistent disruption of the microbiota, as indicated by the long-lasting depletion of beneficial genera, such as *Ligilactobacillus*, *Ruminococcaceae*, and *Lachnospiraceae*, along with sustained overgrowth of *Escherichia-Shigella*. This pattern is consistent with the clinical literature, which shows that clindamycin can eradicate up to 90% of normal gut microbial communities and cause dysbiosis that lasts for months to years [38,39], with slow or incomplete recovery and an increased carriage of antimicrobial resistance genes [40]. The use of clindamycin is associated with several adverse effects, such as diarrhea, abdominal pain, fever, and hematochezia. These side effects may promote the overgrowth of pathogenic bacteria, particularly *Clostridium difficile*, potentially leading to pseudomembranous colitis [41,42], and explain why clindamycin is strongly associated with intestinal inflammation and *C. difficile*-associated disease in humans. These findings suggest that clindamycin may not be the optimal antibiotic choice for penicillin-allergic patients with dental infections, highlighting the need for careful selection of antibiotic regimens [43].

Azithromycin produced moderate decreases in microbial richness during treatment but showed rapid recovery after antibiotic cessation. This reversible profile aligns with epidemiological studies, which report that macrolides induce less persistent microbiota disruption than lincosamides and many β-lactams. However, transient reductions in *Bifidobacterium* and *Lactobacillus* are well-documented. One of clinical evidence shows that azithromycin can reduce the abundance of *Bifidobacterium* and *Lactobacillus* while increasing the levels of *Escherichia coli* [44,45]. A study on Finnish children aged 2–7 years revealed that macrolide antibiotics decreased the relative abundance of Actinobacteria and increased that of Proteobacteria and Verrucomicrobia. Compared to amoxicillin, azithromycin achieves a concentration in gingival crevicular fluid that is approximately 40 times higher than its serum concentration, with sustained levels lasting up to two weeks [46]. In a study by Escalante et al., azithromycin administered before implant surgery exhibited a longer duration of action at the surgical site compared to amoxicillin [47], with higher bioavailability than both amoxicillin and clindamycin [48]. Furthermore, patients treated with azithromycin had lower levels of pro-inflammatory cytokines and chemokines in gingival crevicular fluid and peri-implant crevicular fluid compared to those treated with amoxicillin. Collectively, these findings suggest that azithromycin may be more effective than amoxicillin and clindamycin in promoting the resolution of postoperative inflammation and facilitating early wound healing.

Across all three antibiotics, reductions in SCFA levels closely mirrored the degree of microbiota disruption. Amoxicillin and azithromycin caused moderate decreases in acetate and other major SCFAs, and these changes were partially restored during recovery. Clindamycin caused the most severe changes, with no significant recovery across all time points. These findings are consistent with previous studies that have shown SCFA depletion following the loss of anaerobic fermenters, particularly Ruminococcaceae and Lachnospiraceae, after exposure to broad-spectrum antibiotics [49]. Reduced SCFA availability is known to impair colonocyte metabolism [50,51,52], increase luminal oxygen tension, and promote the overgrowth of facultative anaerobes [53,54], such as Enterobacteriaceae [55,56], a pattern also evident in our absolute quantification sequencing results.

Goblet cell loss and reduced *Muc2* expression followed a similar antibiotic-specific pattern. Amoxicillin caused moderate but reversible reductions in goblet cells and *Muc2* expression, while azithromycin caused no significant reductions. In contrast, clindamycin caused the most profound and persistent reductions. These findings align with mechanistic studies showing that antibiotic-induced dysbiosis alters microbial-associated molecular patterns, reduces SCFA-mediated trophic support, and disrupts epithelial barrier function [26]. Persistent mucus layer deterioration caused by clindamycin may predispose hosts to intestinal inflammation and susceptibility to pathogens, further emphasizing the clinical relevance of antibiotic-specific intestinal toxicity.

The differential impacts of amoxicillin, azithromycin, and clindamycin on gut homeostasis originate from their distinct pharmacological properties and antibacterial spectra. In terms of antibacterial spectrum and target specificity, amoxicillin acts on Gram-positive and some Gram-negative bacteria by disrupting their cell walls, which results in a broad-spectrum disturbance of the gut microbiota. Clindamycin has a strong effect on gut anaerobes, which are crucial for SCFA production and mucus barrier maintenance, thus explaining its sustained suppression of SCFAs and goblet cell function. Azithromycin inhibits bacterial protein synthesis and has weak activity against anaerobic commensals, leading to milder and reversible perturbations. Regarding pharmacokinetics and intestinal concentration, clindamycin is well absorbed, accumulates in intestinal tissues, and has a longer intestinal retention time than amoxicillin or azithromycin, which underpins its persistent effects. Azithromycin, mainly excreted through bile, has weaker direct impacts on the gut microbiota.

The comparative effects of oral antibiotics on gut homeostasis are directly relevant to clinical decision-making in dentistry, where amoxicillin, clindamycin, and azithromycin remain the most frequently prescribed medications. Current ADA and international guidelines recommend amoxicillin as first-line therapy and increasingly favor azithromycin over clindamycin for patients with true penicillin allergy, due to the latter′s strong association with *C. difficile* infection and antimicrobial resistance. The present study provides experimental evidence supporting this shift: azithromycin produced the least disruption to gut microbiota structure, SCFA production, and mucosal integrity, whereas clindamycin produced severe and long-lasting intestinal injury. These findings reinforce the need for cautious use of clindamycin in dental practice and support guideline-based stewardship strategies that emphasize the use of narrow-spectrum agents and the avoidance of high-risk antibiotics.

The findings of this study should be interpreted with consideration of its limitations. As an animal study, the mouse gut microbiome and mucosal immune responses may not fully reflect human physiology. The observation period may also have been too short to capture the whole recovery trajectory after clindamycin, which is known to cause months-long dysbiosis. Though 16S rRNA absolute quantification provides high accuracy for taxonomic abundance, future studies incorporating metagenomics, metabolomics, and host transcriptomics could provide deeper insight into functional changes, resistance gene dynamics, and epithelial responses. Evaluation of post-antibiotic interventions—such as targeted probiotics or dietary SCFA enrichment—may further inform strategies to mitigate intestinal injury following necessary antibiotic therapy.

Taken together, these results demonstrate apparent differences in the intestinal impact of commonly used dental antibiotics, with azithromycin showing the most favorable safety profile and clindamycin the most significant risk for prolonged dysbiosis and mucosal injury. These findings provide mechanistic support for contemporary dental antibiotic stewardship recommendations and emphasize the importance of minimizing unnecessary antibiotic exposure to maintain intestinal homeostasis.

## 4. Materials and Methods

### 4.1. Animals and Experimental Design

This study was reviewed and approved by the Animal Care and Use Committee of Capital Medical University (Ethical Code: AEEI-2023-290) and adhered to the regulations of the People’s Republic of China regarding laboratory animal welfare. Male C57BL/6N mice, aged 6–8 weeks, were housed in a specific pathogen-free environment with a 12 h light/dark cycle at a temperature of 25 ± 2 °C and relative humidity of 50 ± 5%. The mice had free access to standard laboratory chow and water throughout the experimental period.

C57BL/6N mice were purchased from Beijing Vital River Laboratory Animals (Beijing, China). Following a one-week acclimatization period, a total of 120 mice (8 mice per group) were assigned randomly to three time points: antibiotic treatment, one week of antibiotic recovery, and two weeks of antibiotic recovery.

The Amoxicillin based-group (total *n* = 48) included 3 time points, each with 2 subgroups (8 mice/subgroup) [57]. At the 7-day time point, the Control subgroup received 0.9% NaCl (5 μL/g body weight) via oral gavage twice daily for 7 days (10 a.m. and 6 p.m.); and the Amoxicillin (Amx) subgroup received amoxicillin (MedChemExpress, Shanghai, China, HY-B0467) at 307.5 mg/kg/day (30.75 mg/mL, 5 μL/g body weight) via oral gavage twice daily for 7 days (10 a.m. and 6 p.m.), with dosage mimicking clinical use [58]. At 7 days-R1W and 7 days-R2W, the two subgroups received the same 7-day interventions as the 7 days cohort, followed by 1 week and 2 weeks of recovery (no treatment), respectively. (2) For azithromycin and Clindamycin-based groups (total *n* = 72), 3 time points were set, each with 3 subgroups (8 mice/subgroup). At the 5-day time point, the Control subgroup received 0.9% NaCl (5 μL/g body weight) via oral gavage twice daily for 5 days (10 a.m. and 6 p.m.); the Azithromycin (Azit) subgroup received azithromycin (MedChemExpress, Shanghai, China,, HY-17506A) via oral gavage once daily at 10 a.m. (102.5 mg/kg on day 1, 51.25 mg/kg from days 2–5; 10 μL/g body weight, with concentrations 10.25 mg/mL and 5.125 mg/mL respectively); and the Clindamycin (Clin) subgroup received clindamycin (MedChemExpress, Shanghai, China,, HY-B1358) at 246 mg/kg/day (24.6 mg/mL, 5 μL/g body weight) via oral gavage twice daily for 5 days (10 a.m. and 6 p.m.). At 5 days-R1W and 5 days-R2W, the three subgroups received the same 5-day interventions as the 5 days cohort, followed by 1 week and 2 weeks of recovery (no treatment), respectively. All mice were euthanized at the designated time points.

### 4.2. Sample Collection

The cecum weight was measured after sacrifice, and the cecal index was calculated using the formula: cecal index = [cecum weight (mg)/mouse weight (g)] × 100% [59]. Fecal characteristics of mice were recorded every other day. Diarrhea severity was classified into five grades based on stool consistency [60]: 0, normal; 1, slightly wet; 2, moderate moisture; 3, loose; 4, watery stool. Fecal water content was determined using the following formula: (FW − DW)/FW × 100%, where FW represents the weight of fresh feces and DW represents the dry weight of feces obtained through drying at 105 ± 2 °C [61]. Feces and cecal contents were collected immediately and stored at −80 °C. The colon was exposed and excised by cutting from the cecum–colon junction to the upper end of the rectum, yielding an approximately 5 cm long colon segment. Subsequently, the colon was flushed with ice-cold phosphate-buffered saline to remove luminal contents. The proximal segment of the colon, measuring approximately 2.5 cm, was frozen in liquid nitrogen for subsequent analysis. Meanwhile, the distal segment, also around 2.5 cm in length, was fixed for Alcian blue staining.

### 4.3. Microbial DNA Extraction and Accu16STM Assay

The impact of different antibiotics on the fecal microbiome composition in mice was assessed at the phylum and genus levels using precise 16S rRNA absolute quantification sequencing. Microbial DNA extraction and the Accu16STM assay (Accurate 16S Absolute Quantification Sequencing) was conducted by Genesky Biotechnologies Inc. (Shanghai, China). In brief, total genomic DNA was extracted from samples using the FastDNA^®^ SPIN Kit (MP Biomedicals, Santa Ana, CA, USA) according to the manufacturer’s instructions. The integrity of the extracted genomic DNA was evaluated through agarose gel electrophoresis, while its concentration and purity were determined using the Nanodrop 2000 (Thermo Fisher Scientific, Wilmington, DE, USA) and Qubit 3.0 Spectrophotometer (Invitrogen, Carlsbad, CA, USA), respectively. Artificially synthesized spike-ins were designed to share identical conserved regions with natural 16S rRNA genes, while their variable regions were substituted with random sequences exhibiting approximately 40% GC content. A precisely controlled mixture of these spike-ins, featuring known gradient concentrations and predetermined proportions, was subsequently spiked into the genomic DNA sample. The V3–V4 hypervariable regions of the 16S rRNA gene and spike-ins were amplified using primers 341F (5′-CCTACGGGNGGCWGCAG-3′) and 805R (5′-GACTACHVGGGTATCTAATCC-3′) and sequenced on an Illumina NovaSeq 6000 sequencer (Illumina, San Diego, CA, USA). Raw sequencing data were processed using QIIME2 [62]. Adaptor and primer sequences were trimmed using the cutadapt plugin, and quality control and identification of amplicon sequence variants (ASVs) were performed using the DADA2 plugin [63]. Taxonomic assignments of ASV representative sequences were conducted with a confidence threshold of 0.8 using a pre-trained Naive Bayes classifier based on Greengenes (version 13.8). Spike-in sequences were identified, and their read counts were recorded. A standard curve was generated for each sample by correlating read counts with spike-in copy numbers. The absolute copy number of each ASV in every sample was calculated using this standard curve based on its corresponding read count. Spike-in sequences, being distinct from the microbial community in the samples, were excluded from subsequent analyses [64].

### 4.4. Analysis of SCFAs

The procedures outlined by Zhang et al. [65] were followed with slight modifications for sample pretreatment. Approximately 100–400 mg of cecal contents were weighed and mixed with 1.5 mL of 1 M hydrochloric acid (Beijing chemical works, Shanghai, China, H014570.214). The samples were followed by vortexing for 1 min and thorough mixing until no visible clumps remained. The well-mixed centrifuge tube was placed in a beaker containing an ice-water bath, and ultrasonic extraction was carried out for 10 min at room temperature and a frequency of 40 kHz. The sample was then centrifuged at 4 °C and 9710× *g* for 15 min. Subsequently, 1 mL of supernatant was transferred to a new tube, and 1 mL of ethyl acetate (RHAWN, Shanghai, China, R003520) was added. The mixture was inverted several times to ensure complete mixing, followed by another centrifugation under the same conditions for an additional 15 min. The supernatant was aspirated using a disposable sterile syringe and filtered through a 0.22 μm organic filter membrane. Finally, the filtered supernatant was transferred into a clear short-thread wide-mouth sample vial for analysis. Quantification of SCFAs was carried out using gas chromatography–mass spectrometry (GC-MS) with external calibration based on standard curves. Standard curve of acetic acid (Aladdin, Shanghai, China, A116165), propionic acid (MACKLIN, Shanghai, China, P816182), butyric acid (MACKLIN, Shanghai, China, B802730), isobutyric acid (Aladdin, Shanghai, China, I103521), valeric acid (Aladdin, Shanghai, China, V108271), and isovaleric acid (Aladdin, Shanghai, China, I108280) were prepared in accordance with the Zhang’ protocol. All samples were analyzed using an Agilent 7000C triple quadrupole GC-MS/MS system (Agilent Technologies, Santa Clara, CA, USA) coupled with an Agilent 7890B gas chromatograph, and data were processed via Agilent MassHunter Workstation Software (Version B.07.03.2129, Agilent Technologies, Santa Clara, CA, USA) The chromatographic conditions were as follows: the inlet temperature was set to 230 °C, high-purity helium was used as the carrier gas, and the column flow rate was maintained at 1.2 mL/min. The injection volume was 1 µL, with a split ratio of 9:1. The chromatographic column was purchased from Agilent (Agilent Technologies, Santa Clara, CA, USA G3903-63008, 30 m × 0.250 mm × 0.25 µm). The temperature gradient program was as follows: the initial temperature was held at 120 °C for 2.5 min, then increased to 130 °C at a rate of 8 °C/min and held for 4 min, followed by an increase to 210 °C at a rate of 10 °C/min and held for 5 min. Mass spectrum conditions were set as follows: the ionization mode utilized electron bombardment ion source (EI) with 70 eV electron bombardment energy and an ion source temperature of 230 °C. The mass spectrometric data were acquired in scan mode over a mass range of 10 to 150 *m*/*z*, with a 4 min solvent delay time.

### 4.5. Alcian Blue Staining

The colon tissues were fixed in 4% (*w*/*v*) paraformaldehyde for 24 h, subsequently dehydrated through a graded series of ethanol solutions, embedded in paraffin, and sectioned into slices with a thickness of 5 µm. For the quantification of goblet cells, the tissue sections were stained following the manufacturer’s instructions with Alcian Blue solution (Servicebio, Wuhan, China, G1560).

### 4.6. Real-Time Quantitative PCR

Real-time quantitative PCR (RT-PCR) was used to determine the relative mRNA levels of *Muc2*. Total RNA was extracted from the colon tissue using Trizol (Invitrogen, Carlsbad, CA, USA, 15596018CN), followed by reverse transcription into cDNA with FastKing gDNA Dispelling RT SuperMix (Tiangen, Beijing, China, KR118). Real-time quantitative PCR was performed using a real-time fluorescent quantitative PCR system (Bio-Rad, Hercules, CA, USA) with SYBR Green Premix pro TaqHS qPCR kit (Accurate Biotechnology, Changsha, China, AG11739). The primer sequences are detailed in Table 1. The reaction conditions included an initial denaturation at 95 °C for 30 s, followed by PCR at 95 °C for 5 s and 60 °C for 30 s, cycled 35 times to determine Ct values for *Muc2* gene and β-actin. Gene expression was quantified using the 2^−ΔΔCt^ method (Table 1).

### 4.7. Statistical Analysis

Statistical analysis involved assessing data distribution normality using the Shapiro–Wilk test. Intergroup comparisons were made using independent two-sample *t*-tests under the assumption of normal variance. When assumptions were violated, the non-parametric Mann–Whitney U test was used. For 16S rRNA sequencing data analysis, the Mann–Whitney U test was applied between two independent groups. All statistical analyses were performed using SPSS version 23.0 for Windows. Graphs were created with GraphPad Prism 8.0 software (GraphPad Software, Inc., Boston, MA, USA). Statistical significance was denoted by * *p* < 0.05, ** *p* < 0.01, and *** *p* < 0.001 compared to the control group.

## 5. Conclusions

Within the limits of this study, amoxicillin, azithromycin, and clindamycin have distinctly different effects on intestinal homeostasis when administered at clinically relevant doses. Amoxicillin caused moderate yet partially reversible disturbances in gut microbiota composition, SCFA production, and colonic goblet cell function. Azithromycin produced only mild and transient alterations across all measured parameters, indicating a relatively favorable intestinal safety profile. In comparison, clindamycin caused the most severe and persistent dysbiosis, characterized by a significant expansion of Proteobacteria, depletion of beneficial taxa, reductions in SCFA levels, and a long-lasting impairment of goblet cells and *Muc2* expression, without recovery during the observation period. These findings provide experimental evidence that commonly used dental antibiotics differ substantially in their effects on the gut environment. In contexts where antibiotics are clinically indicated, particularly for patients with true penicillin allergy, azithromycin may represent a microbiota-sparing alternative to clindamycin. The severe intestinal impact of clindamycin underscores the importance of careful stewardship and adherence to current prescribing guidelines. Further studies integrating metagenomic, metabolomic, and longer-term follow-up analyses are warranted to characterize recovery trajectories better and develop strategies for mitigating antibiotic-associated intestinal dysbiosis.

## Figures and Tables

**Table 1:** RT-PCR primer sequences.

Genes	Forward Primer (5′-3′)	Reverse Primer (5′-3′)
*M* *uc* *2*	TGCTGACGAGTGGTTGGTGAATG	TGATGAGGTGGCAGACAGGAGAC
*β-actin*	GGCTGTATTCCCCTCCATCG	CCAGTTGGTAACAATGCCATGT

## Data Availability

All data generated or analyzed during this study are included in this article. Further inquiries can be directed to the corresponding authors.

## Supplementary Materials

The following supporting information can be downloaded at: https://www.mdpi.com/article/10.3390/antibiotics15010024/s1, Figure S1: Microbial community composition at the phylum level in the amoxicillin, azithromycin, and clindamycin groups; Figure S2: Key Bacterial Genera in the Fecal Microbiota of C57BL/6N Mice After Treatment with Amoxicillin, Azithromycin, and Clindamycin; Figure S3: Effects of Amoxicillin, Azithromycin, and Clindamycin on the SCFAs in C57BL/6N Mice.

## Abbreviations

The following abbreviations are used in this manuscript:

SCFAs	short-chain fatty acids
GC-MS	gas chromatography–mass spectrometry
EI	ion source
