# Supplementary material for: Comparative Impacts of Oral Amoxicillin, Azithromycin, and Clindamycin on Gut Microbiota and Intestinal Homeostasis
Source: Antibiotics (Basel). 2025 Dec 25;15(1):24. doi: 10.3390/antibiotics15010024 (PMC12837590; doi:10.3390/antibiotics15010024)
Supplement: Supplementary file 1 [file antibiotics-15-00024-s001.zip › antibiotics-4039352-supplementary.pdf]

Supplementary Information

Comparative Impacts of Oral Amoxicillin, Azithromycin, and Clindamycin on Gut Microbiota and Intestinal Homeostasis

Shanshan Li [1,2], Jing Sun [2,3,4], Yanfang Ren [5,*] and Songlin Wang [2,4,6,*]

[1] School of Stomatology, Henan University, Zhengzhou 450046, China; shanshanli@henu.edu.cn
[2] Laboratory of Oral Health and Homeostatic Medicine, School of Stomatology and Beijing Laboratory of Oral Health, Capital Medical University, Beijing 100070, China; sunjing6721@163.com
[3] Central Laboratory, Jinan Key Medical and Health Laboratory of Oral Diseases and Tissue Regeneration, Jinan Key Laboratory of Oral Diseases and Tissue Regeneration, Shandong Provincial Key Medical and Health Laboratory of Oral Diseases and Tissue Regeneration, Jinan Stomatological Hospital, Jinan 250001, China
[4] Department of Biochemistry and Molecular Biology, School of Basic Medical Sciences, Capital Medical University, Beijing 100069, China
[5] Eastman Institute for Oral Health, University of Rochester Medical Center, 625 Elmwood Ave., Rochester, NY 14620, USA
[6] Laboratory of Oral Homeostatic Medicine, School of Medicine and SUSTech Homeostatic Medicine Institute (SHMI), Southern University of Science and Technology, Shenzhen 518055, China
***** Correspondence: yanfang_ren@urmc.rochester.edu (Y.R.); slwang@ccmu.edu.cn (S.W.)

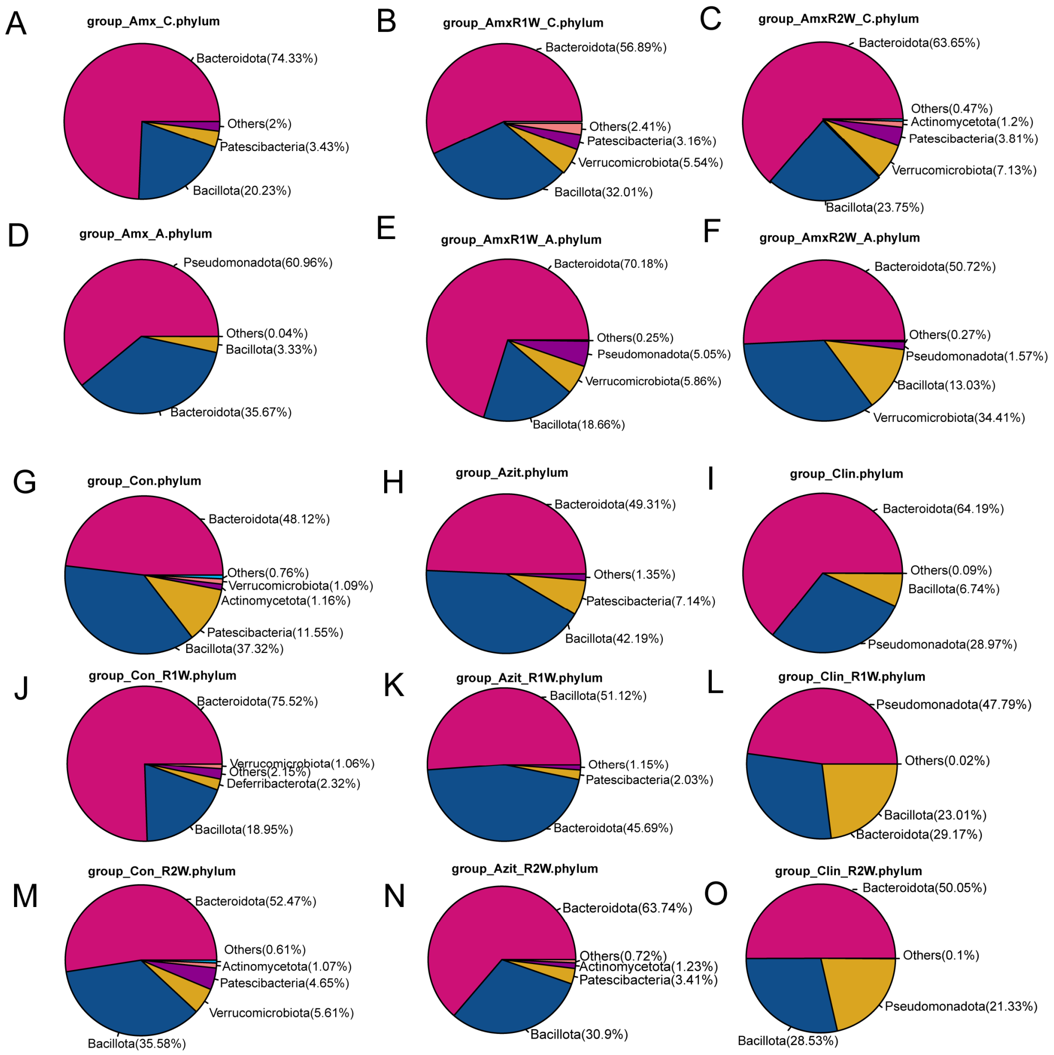

Figure S1. Microbial community composition at the phylum level in the amoxicillin, azithromycin, and clindamycin groups. The pie plot displays the microbial community composition at the phylum level for the amoxicillin group (A-F), azithromycin group, and clindamycin group (G-O) across various time points(n = 8).

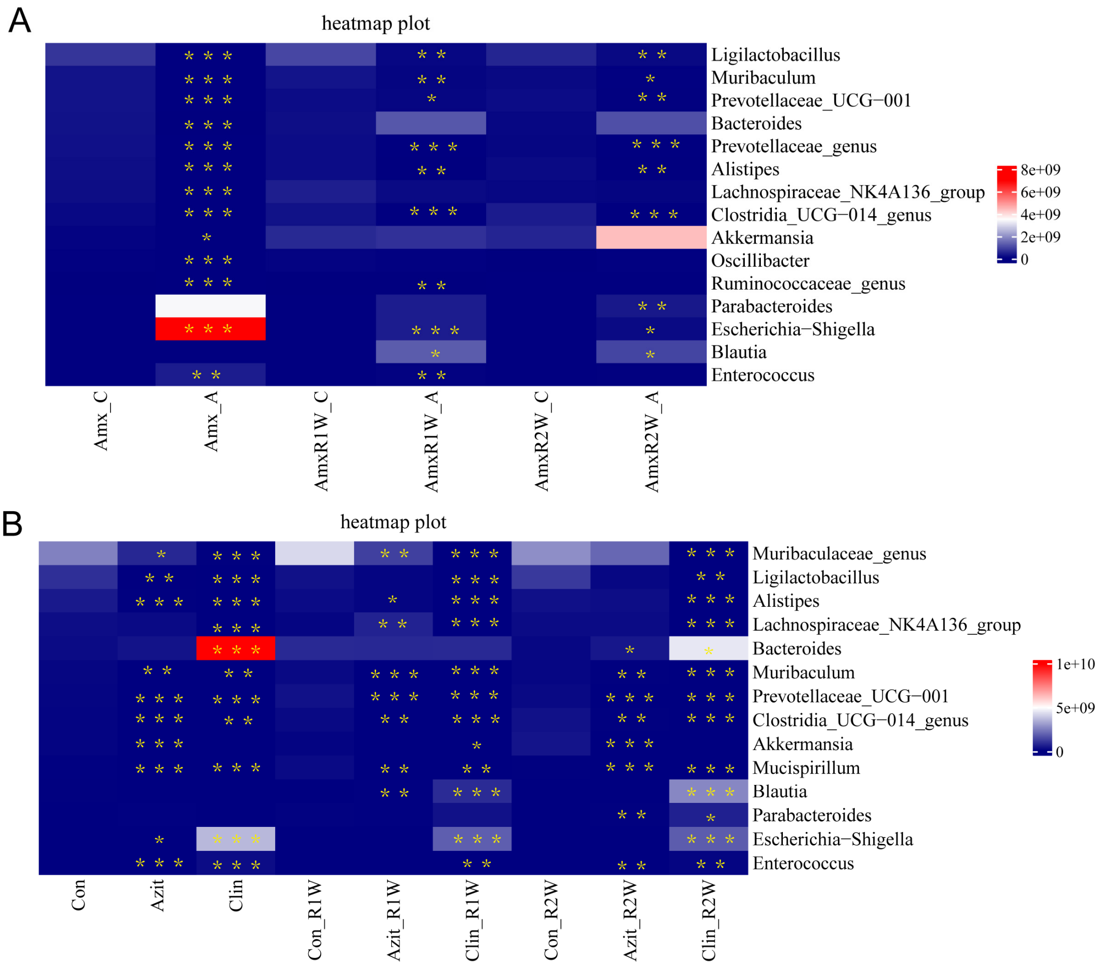

Figure S2. Key Bacterial Genera Present in the Fecal Microbiota of C57BL/6N Mice following Treatment with Amoxicillin, Azithromycin, and Clindamycin. The heatmap illustrates the average absolute abundance of key bacterial genera that demonstrated significant variation across samples from the amoxicillin, azithromycin, and clindamycin treatment groups by Accurate 16S Absolute Quantification Sequencing. Similar to the trend observed for *Escherichia-Shigella* in the amoxicillin group, the levels of Parabacteroides and *Enterococcus* increased in the Amx_A group and subsequently decreased gradually over time, while the level of *Akkermansia* showed a gradual increase throughout the observation period(A). The level of *Blautia* increased in the group treated with Clindamycin over time. Data are presented as mean ± SD (n = 8). *p < 0.05, **p < 0.01, ***p < 0.001 compared with the control group.

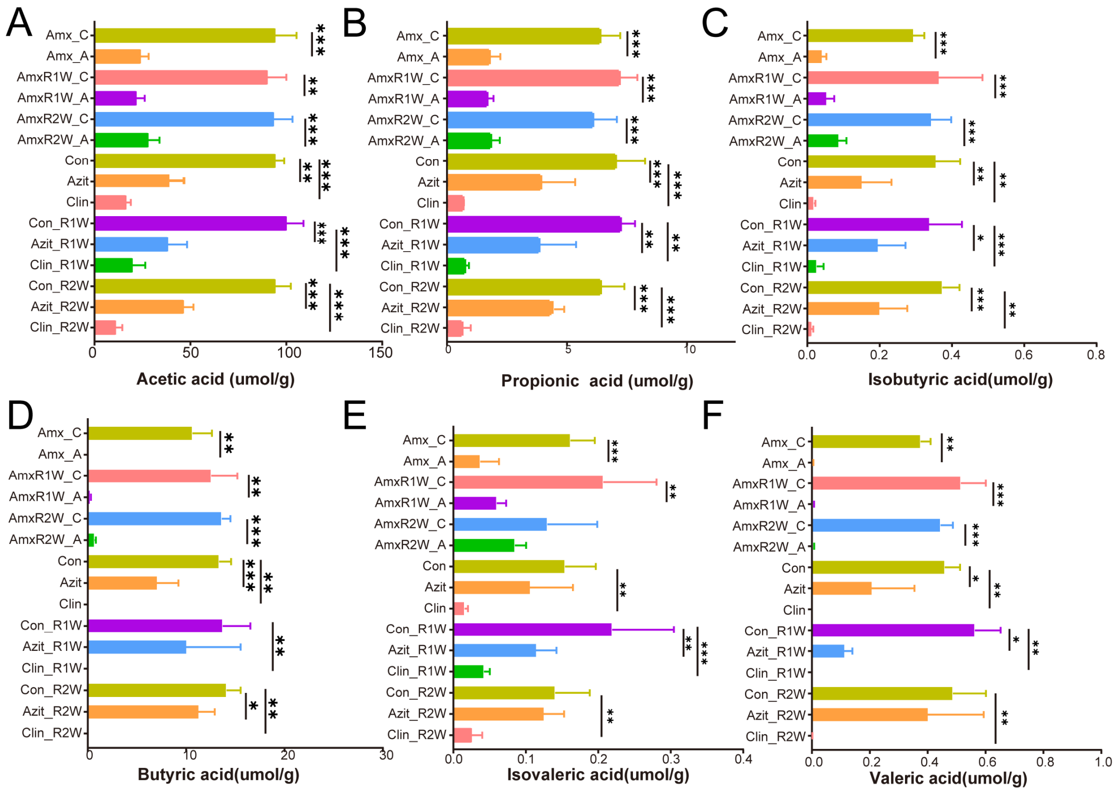

Figure S3: Impact of Amoxicillin, Azithromycin, and Clindamycin on SCFAs in C57BL/6N Mice. GC-MS analysis was conducted to assess SCFAs levels in cecal content post administration of the antibiotics.Amoxicillin significantly reduced the levels of acetic acid, propionic acid, isobutyric acid, butyric acid, isovaleric acid, valeric acid, and total SCFAs in cecal contents at various time points, except for isovaleric acid in the AmxR2W group. In mice treated with azithromycin, SCFAs levels were significantly lower than those in the respective control groups, except for butyric acid in the AmxR2W group. The clindamycin group exhibited a substantial decrease in SCFAs levels at all time points, which was more severe compared to both the amoxicillin and azithromycin groups (A-F). Con, control group; Amx, amoxicillin group; Azit, azithromycin group; Clin, clindamycin group. Data are expressed as mean ± standard deviation (n = 6). *$p < 0.05$, **$p < 0.01$, ***$p < 0.001$ compared with the control group.
